# Estimation of Surface Radiation Dosage to Thyroid Gland and Lower Abdomen While Using Intraoral Periapical Radiography: A Phantom Study

**DOI:** 10.7759/cureus.19727

**Published:** 2021-11-18

**Authors:** Krishnamachari Janani, T Malarkodi, Sathasivasubramanian Sankarapandian

**Affiliations:** 1 Department of Conservative Dentistry and Endodontics, SRM Dental College, SRM Institute of Science and Technology, Ramapuram, Chennai, IND; 2 Oral Medicine and Radiology, Sri Ramachandra Institute of Higher Education and Research, Chennai, IND

**Keywords:** thyroid and lower abdomen, radiation dose, thermoluminescent dosimeter, surface radiation dose, intraoral periapical radiography

## Abstract

Background and objective

Every dose of radiation has the potential to cause biological harm. Quantitative assessment of radiation doses to radiosensitive organs can aid dental professionals in taking the appropriate protective measures against radiation. This data may also be used among the general public to alleviate fears of radiation exposure in dental radiography. This study aimed to estimate the surface radiation dose at the level of the thyroid and lower abdomen during intraoral periapical radiography (IOPAR).

Materials and methods

A total of 80 calcium sulfate (CaSO_4_) discs were utilized in this cross-sectional in vitro study to estimate the surface radiation dose at the level of thyroid and lower abdomen on a phantom model while using an IOPAR. After exposure, the discs were submitted to the Radiation Laboratory's "Personnel Monitoring Service" to measure the surface radiation dose. Mean and standard deviations were calculated using descriptive statistics for continuous variables. The Mann-Whitney U test was used to compare bi-variate samples of independent groups. All statistical analyses were performed using SPSS Statistics version 21.0 (IBM, Armonk, NY).

Results

The results showed a statistically significant difference in radiation exposure between the maxillary anterior and mandibular anterior regions when the thermoluminescent dosimeter (TLD) was placed in the lower abdomen (p=0.000). When the maxillary and mandibular posterior regions were compared, there was a statistically significant difference in radiation exposure when the TLD was placed in the lower abdomen (p=0.000).

Conclusion

When the cone was positioned in the maxillary region, there was an increase in surface radiation dosage to the lower abdomen and thyroid.

## Introduction

An intraoral periapical radiograph (IOPAR) is one of the most significant and commonly used diagnostic methods for a variety of dental pathologies [1]. Despite its major role in dental imaging, dentists are concerned about the adverse effects of ionizing radiation. To protect oneself from the hazardous effects of radiation, which causes somatic alterations and genetic mutations, radiation protection measures are mandatory. Exposure to radiation can be reduced by following proper techniques when taking radiographs, resulting in fewer radiographic examinations and less radiation exposure [2]. Medical X-ray exposure accounts for 14% of the population's annual dose. More than 95% of human exposure to artificial radiation comes from diagnostic and interventional radiology, making it a risk factor for cancer [3]. 

Dental radiography is the prevailing imaging technique in dentistry according to the United Nations Scientific Committee on the Effects of Atomic Radiation (UNSCEAR) 2000 report, and it continues to play a crucial role in daily dental practice. Despite the fact that dental X-ray machines emit a small amount of radiation, they can still cause stochastic effects [4]. Dental radiography investigations account for 25.25% of all radiographic examinations conducted worldwide. The radiation dosage should always be measured since it is an extremely important quality tool in dental radiology. The Diagnostic Reference Level (DRL) has been set at 7 mGy by the International Commission on Radiation Protection (ICRP) [5].

When compared to other dental specialties, endodontists use IOPAR more frequently. A minimum of three to four radiographs is required to accomplish one root canal procedure. An endodontist who treats a minimum of five patients per day is exposed to radiation about 20 times. The focus of this research was to use thermoluminescent dosimeter (TLD) discs to determine the surface radiation exposure at the level of the thyroid and lower abdomen on a phantom model.

## Materials and methods

After obtaining ethical approval from the Institutional Ethics Committee (IRB No. CSP/14/APR/32/53), an in vitro study was designed. The study was performed on a phantom model with no human subject involvement. The dose was measured with a TLD disc [calcium sulfate (CaSO4)]. The phantom head was held in a position similar to that of a patient. To portray this feature in radiography, a phantom with similar density material to the human body was chosen. The entire procedure was carried out in a 100-square-foot radiography room with a 30-centimeter wall thickness, a door with leaded glass, and a 1.7-inch-thick lead lining. A protective lead barrier screen was used, along with a unit controlled remotely. Personnel involved in the study were restricted and not exposed to radiation at any time during the study. The complete assembly was inspected for radiation safety prior to the study to ensure that the equipment was working properly and the radiation protection measures in place were adequate.

The materials used were as follows: intraoral x-ray machine (Satelec X Mind AC 2007), TLD discs, and a phantom model. TLD discs were secured on the phantom model with tape. A total of 80 CaSO_4_ discs were used - Group 1: 40 discs were used for the thyroid region: Group 2: 40 discs were used for the lower abdomen. 

Subgroups for TLD discs placed in the thyroid region were as follows: 

Group 1a: maxillary anterior (10 discs), Group 1b: maxillary posterior (left) (10 discs), Group 1c: mandibular anterior (10 discs), Group 1d: mandibular posterior region (right) (10 discs). Similar to the above-mentioned subgroups, 40 TLD discs were placed on the lower abdomen region. Group 2a: maxillary anterior (10 discs), Group 2b: maxillary posterior (left) (10 discs), Group 2c: mandibular anterior (10 discs), Group 2d: mandibular posterior region (right) (10 discs).

For 20 exposures, one disc at the level of the thyroid and one disc at the level of the lower abdomen were simultaneously exposed with the cone positioned in the maxillary anterior area. Similarly, 20 exposures were made with the cone positioned at the maxillary posterior (left), mandibular anterior (right), and mandibular posterior (left). Each TLD disc was exposed 20 times in total. The exposure period was set to 0.8 seconds, the tube voltage was set to 70 kVp, and the tube current was set to 8 mA, and the surface radiation doses for the thyroid and lower abdomen were measured at the same time.

Statistical analysis

The data were analyzed using statistical tests. For continuous variables, descriptive statistics were used to calculate mean and standard deviations. To compare bi-variate samples of independent groups, the Mann-Whitney U test was utilized. All statistical analyses were performed using SPSS Statistics version 21.0 (IBM, Armonk, NY).

## Results

The surface radiation dose at the level of the lower abdomen was determined to be 3.5 mSv when the cone was positioned in the maxillary anterior region, 3.5 mSv in the maxillary posterior (left) region, 0.09 mSv in the mandibular anterior region, and 0.05 mSv in the mandibular posterior (right) region. When the TLD was positioned in the lower abdominal region, the results demonstrated a statistically significant difference between the maxillary anterior and mandibular anterior regions (p=0.000) (Table 1).

**Table 1:** Surface radiation in the lower abdomen for maxillary and mandibular anterior teeth

Groups	N	Mean	Standard deviation	P-value
Maxillary anterior	10	3.5760	0.49862	0.000
Mandibular anterior	10	0.0960	0.03098	

The radiation exposure to the lower abdomen was greater when the cone was positioned in the maxillary posterior than when the cone was positioned in the mandibular posterior, and this difference was statistically significant (p=0.000) (Table 2).

**Table 2:** Surface radiation in the lower abdomen for maxillary and mandibular posterior teeth

Groups	N	Mean	Standard deviation	P-value
Maxillary posterior	10	3.5010	0.41089	0.000
Mandibular posterior	10	0.0560	0.02319	

When the cone was positioned in the maxillary anterior region, the mean surface radiation dose at the level of the thyroid was 4.13 mSv, 3.82 mSv for the maxillary posterior (left) region, 3.17 mSv for the mandibular anterior region, and 0.45 mSv for the mandibular posterior (right) region. In the case of the thyroid, the maximum surface radiation dose was detected in the maxillary, mandibular anterior (Table 3), and maxillary posterior region. When the overall significance was calculated, all of the groups had a statistically significant difference with a p-value of less than 0.05 (Table 4).

**Table 3:** Surface radiation in the thyroid region for maxillary and mandibular anterior teeth

Groups	N	Mean	Standard deviation	P-value
Maxillary anterior	10	4.13	0.47	0.008
Mandibular anterior	10	3.17	1.40	

**Table 4:** Surface radiation in the thyroid region for maxillary and mandibular posterior teeth

Groups	N	Mean	Standard deviation	P-value
Maxillary posterior	10	3.82	0.22	0.0002
Mandibular posterior	10	0.45	0.22	

## Discussion

The most common radiograph taken for regular dental assessment is IOPAR. It is impossible to underestimate the relevance of IOPAR in detecting pulp and periapical pathology [6]. Clinical problems that necessitate further investigation include the discovery of a missing canal or extra canal, canal calcification, root resorption management, and vertical root fracture, all of which necessitate a cone-beam CT (CBCT) [7,8]. A root canal treatment, for example, demands a maximum of three radiographs be performed (preoperative radiograph, master cone, and postoperative radiograph). Though the use of electronic apex locators has reduced the necessity for numerous radiographs, they cannot be entirely avoided.

The use of IOPAR for the full mouth revealed 1.5X10(-2) µGy of surface radiation dose and 1.5 mSv of effective dose exposure in the gonadal region [9,10]. A full-mouth examination employing a photostimulable phosphor sensor resulted in an effective dosage of 15 mSv in one study [11], while in another, it was reported to be 34.9 mSv [12].

Gibbs [13] calculated thyroid gland exposure levels as high as 0.49 mSv during a full-mouth examination utilizing IOPAR. The radiation exposure to the thyroid using 16-22 IOPAR ranged from 400 µGy to 920 µGy, according to Gibbs and Danforth and Clark [13,14]. Sheikh et al. [9] estimated the surface radiation dose to the thyroid and gonads using 10 IOPAR surveys to be 10.93 mRads and 0.4 mRads, respectively. The surface dosage for intraoral radiography was calculated to be 2.89 ±2.12 mGy, with a median of 2.43 mGy and the 75th percentile of 3.37 mGy, according to González et al. [10]. On a phantom, Yakoumakis et al. [15] observed a mean entrance surface dose of 3.8 mGy. Using TLD badges, Mortazavi et al. [5] determined the entrance surface radiation dose to the skin at the maxillary and mandibular region as 1.173 ±0.616 mGy.

The overall area of exposure is reduced by limiting the X-ray beam size, which helps to prevent exposing sensitive parts outside the area being imaged for dental purposes, such as the lens of the eye or the thyroid gland. In clinical use, decreasing the X-ray beam diameter optimizes image diagnostic quality by reducing the amount of fogging generated by scattered radiation [16]. A rectangular collimator that confines the X-ray beam to a size just larger than the dental image receptor used is particularly desirable. As a result, lowering the beam size reduces scattered radiation while also improving image quality [17]. The filter material used in X-ray tubing has a significant role in penetrating radiation. Filter material made of aluminum or material equivalent to aluminum tends to absorb less penetrating radiation. Exposure time and tube voltage have a role in minimizing the radiation dosage. When the kilovoltage peak is more than 90, it can cause deleterious effects [18]. Moreover, beam size and the exposure factors are said to vary from one machine to another, which also needs to be taken into consideration.

It is indispensable and mandatory to follow safety protocols to prevent being exposed to scattered radiation. The thickness of the lead-impregnated apron must be within the range of 0.25 to 0.5 mm [19]. Reports have shown almost 85% reduced exposure following the use of lead barriers. As this was a preliminary study, it was carried out on a phantom model. The limitations of the study include the fact that it was conducted on a phantom model; however, tissue-equivalent anthropomorphic phantom models and lithium sulfate TLD discs can be used to estimate absorbed doses.

## Conclusions

This phantom study proved to be a trial for quantifying radiation dosage at the surface. When the cone was positioned in the maxillary region, the surface radiation dose to the thyroid and abdomen increased. Our key takeaway from this study is that it is mandatory to use a thyroid collar and lead apron on any patient who needs an IOPAR, and more so in cases of maxillary teeth as the X-ray beam is directly targeted at these regions. This study may be useful for dental practitioners worldwide to adopt necessary preventative measures to limit radiation exposure and alleviate patients’ concerns about dental radiography.
